# Comparative Cytotoxicity Study of Silver Nanoparticles (AgNPs) in a Variety of Rainbow Trout Cell Lines (RTL-W1, RTH-149, RTG-2) and Primary Hepatocytes

**DOI:** 10.3390/ijerph120505386

**Published:** 2015-05-20

**Authors:** Mona Connolly, Maria-Luisa Fernandez-Cruz, Alba Quesada-Garcia, Luis Alte, Helmut Segner, Jose M. Navas

**Affiliations:** 1 Instituto Nacional de Investigación y Tecnología Agraria y Alimentaria (INIA), Carretera de la Coruña Km 7.5, E-38040 Madrid, Spain; E-Mails: connolly.mona@inia.es (M.C.); fcruz@inia.es (M.-L.F.-C.); quesada.alba@inia.es (A.Q.-G.); luis.alte@inia.es (L.A.); 2 Faculty of Vetsuisse, Centre for Fish and Wildlife Health, University of Berne, Länggassstr. 122, Postfach 8466, CH-3001 Bern, Switzerland, E-Mail: helmut.segner@vetsuisse.unibe.ch

**Keywords:** AgNPs, cytotoxicity, rainbow trout (Oncorhynchus mykiss), *in vitro* cell lines, primary hepatocyte

## Abstract

Among all classes of nanomaterials, silver nanoparticles (AgNPs) have potentially an important ecotoxicological impact, especially in freshwater environments. Fish are particularly susceptible to the toxic effects of silver ions and, with knowledge gaps regarding the contribution of dissolution and unique particle effects to AgNP toxicity, they represent a group of vulnerable organisms. Using cell lines (RTL-W1, RTH-149, RTG-2) and primary hepatocytes of rainbow trout (Oncorhynchus mykiss) as *in vitro* test systems, we assessed the cytotoxicity of the representative AgNP, NM-300K, and AgNO3 as an Ag+ ion source. Lack of AgNP interference with the cytotoxicity assays (AlamarBlue, CFDA-AM, NRU assay) and their simultaneous application point to the compatibility and usefulness of such a battery of assays. The RTH-149 and RTL-W1 liver cell lines exhibited similar sensitivity as primary hepatocytes towards AgNP toxicity. Leibovitz’s L-15 culture medium composition (high amino acid content) had an important influence on the behaviour and toxicity of AgNPs towards the RTL-W1 cell line. The obtained results demonstrate that, with careful consideration, such an *in vitro* approach can provide valuable toxicological data to be used in an integrated testing strategy for NM-300K risk assessment.

## 1. Introduction

Among the plethora of nanoparticles (NPs) already on the market, silver nanoparticles (AgNPs) are being used in more products (>400) than any other manufactured nanomaterial (MN) [1]. AgNPs’ antimicrobial properties, catalytic activity and conductivity have led to their wide application in biomedicine, electronics, the food sector (in packaging materials), and even the textile industry. Mueller and Nowack provided an estimate of a worldwide production of 500 tonnes per annum in 2008, however with continued growth in the industry, this would be significantly higher today [2]. There is evidence to suggest that AgNPs are currently being released into the aquatic environment simply through the use of consumer products containing them [3,4,5]. This, along with the fact that the toxicity of AgNPs has been demonstrated in a range of aquatic organisms [6], with reported LC50s <0.1 mg/L, highlights an environmental risk that warrants careful assessment. According to current environmental risk assessments based on modelling predicted environmental concentrations (PEC) and taking into account species sensitivity distributions, AgNPs present one of the highest risks compared to other NPs, particularly in surface waters [7].

Fish are particularly susceptible to the toxic effects of metal ions such as silver [8,9] that could be released from NPs. There have been significant advancements in the amount of AgNP toxicity studies performed in fish, using for instance zebrafish (Danio rerio) [10,11,12], Japanese medaka (Oryzias latipes) [13,14], sheephead minnow (Cyprinodan variegatus) [15] or rainbow trout (Oncorhynchus mykiss) [16]. The reported LC50 values from these studies were very different ranging from 0.089 µg/mL to 250 µg/mL. This leads to discrepancies in their classification as being toxic or very toxic to the environment according to the European Council directives 67/548/EEC [17] and its further amendment by the Regulation (EC) No 1272/2008 on the classification, labelling and packaging (CLP) of substances and mixtures (directive 2008/112/EC) [18].

Fish cells maintained in primary culture or as immortalized cell lines can represent an economically feasible robust *in vitro* testing system to meet the high number of substances to be tested, including MNs. A large number of fish cell lines exist from different species, tissues and cell types, however they have only been used in a limited number of studies for nanoparticle toxicity assessment [19,20,21,22]. Although such *in vitro* models are yet under validation, information gathered from non-validated *in vitro* systems can still be used in a weight of evidence approach of an integrated testing strategy according to the Commission Regulation (EC) No 134/2009 concerning the Registration, Evaluation, Authorisation and Restriction of Chemicals (REACH) [23]. Ethical reasons and the fulfilment of the 3Rs’ principles (replacement, reduction and refinement) also favour the application of cells in culture. Finally, the ease of maintenance and reproducibility of results makes these systems the ideal tool for studies devoted to determining possible mechanisms underlying the toxicity of chemicals and MNs. However, when using an *in vitro* approach, it is important to carefully consider exposure routes and possible target sites to produce relevant data.

*In vivo* exposures in fish have shown that AgNPs can accumulate in the liver [24,25]. Furthermore, after waterborne exposure to AgNPs, it has been shown that the liver burden of silver was approximately twice that of the gills [26], demonstrating that if the NPs are taken up by fish, they will translocate to the liver as a major site of clearance regardless of the route of exposure. Thus, in the present study, we have employed liver cell lines from rainbow trout, RTH-149 and RTL-W1 as relevant test systems to determine the toxicity of AgNPs *in vitro*. In addition, and for comparative purposes, a rainbow trout gonadal cell line (RTG-2) was also used. Since one of the main limitations of cell lines is the lack of functional receptors or enzymes leading to the disappearance of essential metabolic routes, in this work primary hepatocytes were included to assess differences in sensitivity among continuous cell lines and primary cells whose liver-specific metabolic functions are conserved. Previous studies comparing responses to AgNP exposure in a human hepatocellular cell line and primary hepatocytes showed very good correlation [27].

The main objective of this work was therefore to determine the toxicity of AgNPs in liver cells of rainbow trout as a model system and to shed light on the mechanisms underlying the observed toxic response (if any). This information ultimately would be useful and pertinent in an integrated intelligent testing strategy for AgNP risk assessment. The lack of standardisation for MN testing, confounded by the fact that different MNs have been used with different cappings and coatings, may explain why up until now there have been huge variations in reported toxicities of NPs. To facilitate future comparisons with this study, we have used a representative manufactured silver nanomaterial (NM-300K) which was tested in the testing programme overseen by the OECD Working Party on Manufactured Nanomaterials [28]. As it is still not clear whether the effects of AgNPs are dominated by released silver ions or are caused by the unique properties of the particles themselves, we have assessed in parallel the toxicity of AgNO_3_ which acts as a source of Ag+. In this study, liver cell lines appeared to be a sensitive test system when compared to primary hepatocytes, what has important practical implications taking into account the ease of maintenance and culture of cell lines. The simultaneous use in the same plate of three assay systems evidencing three different mechanisms of toxic action appeared as very appropriate to determine the cytotoxic activity of AgNPs.

## 2. Experimental Section

### 2.1. Chemicals and Reagents

Fetal bovine serum (FBS), ultraglutamine 1 (200 mM), L-glutamine (200 mM), penicillin and streptomycin (P/S) (10,000 U/mL/10 mg/mL), phosphate-buffered saline (PBS), Trypsin/Ethylene diaminetetraacetic acid (EDTA) (17,000 U trypsin/L, 200 mg/l EDTA), non-essential amino acids (NEAA)100X, Eagle's minimal essential medium (EMEM), Minimum Essential Medium (MEM) Alpha (α-MEM) and Leibovitz’s L-15 medium were purchased from Lonza (Barcelona, Spain). Serum-free/phenol red-free MEM (MEM(-)) used was from PAN Biotech, Aidenbach, Germany. Ethanol was from Panreac (Barcelona, Spain). Neutral red (NR, 3-amino-7-dimethylamino-2-methylphenanzine hydrochloride) solution (3.3 g/L), silver nitrate (AgNO_3_), EDTA, sodium pyruvate and bovine serum albumin (BSA) were supplied by Sigma-Aldrich (Madrid, Spain). AlamarBlue® dye and the 5-Carboxyfluorescein diacetate-acetoxymethyl ester (CFDA-AM) probe were from Life Technologies (Madrid, Spain).

For primary hepatocyte isolation Venofix® A perfusion cannulae (15 × 0.5 mm length) (B. Braun Melsungen AG, Melsungen, Germany) were used. Liquid heparin (5000 U.I heparin / 1 mL) was sourced from Drossapharm AG/SA Basel, Switzerland. Ethyl 3-aminobenzoate methanesulfonate anaesthetic (MS-222), collagenase (collagenase IV from Clostridium histolyticum), calcium- and magnesium-free GIBCO® Dulbeccos Phosphate Buffered Saline (DPBS), calcium chloride (CaCl2), and trypan blue (0.4%) solution were supplied by Sigma-Aldrich (Buchs, Switzerland). Sefar PETEX Nylon screens (mesh size 50 µm, 105 µm, 250 µm) were purchased from Sefar AG (Heiden, Switzerland). The peristaltic pump used for perfusion was an ISMATEC® IPC High Precision Multichannel Dispenser and was supplied by IDEX Health & Science (Wertheim, Germany). Medium 199 (M199) (with Earle′s salts, L-glutamine and sodium bicarbonate), used for culturing the primary hepatocytes, was purchased from Sigma Aldrich (Madrid, Spain).

### 2.2. Nanoparticles

The representative silver nanomaterial NM-300K was provided by the European Commission Joint Research Centre (JRC) (Ispra, Italy) in the framework of the FP7 Project MARINA (Managing Risks of Nanomaterials) project. Vials contained 2 mL of an already dispersed AgNP suspension with 10.16% Ag content. Samples were stored at room temperature (~20 ˚C) under dark conditions. Prior to use, vials were shaken vigorously to ensure homogeneity. The exact concentration of nano-silver was measured (mg per mL) according to the handling procedure for weighing and sample introduction outlined in the JRC scientific and technical report on the characterisation, stability and homogeneity of this nanomaterial [29]. Exposure concentrations of the NM-300K dispersion were prepared directly in culture medium with no intermediate dilution in ultrapure water. We were also provided with the NM-300K sample dispersant (NM-300KDIS); an aqueous dispersant with stabilizing agents, consisting of 4% w/w each of polyoxyethylene glycerol trioleate and polyoxyethylene and sorbitan mono-Laurat (Tween 20). This dispersant acted as a vehicle control in all assays to test for any effects from the dispersant only.

### 2.3. Physico-Chemical Characterization of Nanoparticles

Dynamic light scattering (DLS) was applied to characterise the size distribution of AgNPs following suspension (time 0) and, after 24 h incubation in the corresponding cell culture, medium was put under appropriate culture conditions (according to cell line or primary hepatocytes being exposed). A Zetasizer Nano-ZS (Malvern Instruments Ltd., Malvern, UK) was used. Measurements were performed with the highest NM-300K exposure suspension concentrations (93.5 µg/mL). Size distributions are reported according to the mean of three independent measurements per sample, with each measurement consisting of four individual readings, and calculated using Zetasizer Software version 6.34 (Malvern Instruments Ltd). Due to the potential masking of smaller particles by using only intensity measurements, hydrodynamic sizes were also presented according to volume and number weighted distributions taking into account the AgNPs refractive index. The width and average size of NM-300K size distributions were also taken into account according to the polydispersity index (PdI) and Z-average values, respectively. Measurements with only culture media were used as background controls as the presence of large proteins, and other media components will give size distribution readings. The NM-300K dispersion was also diluted in ultrapure Milli-Q water (93.5 µg/mL) to determine the size distribution prior to suspension in culture medium. MEM(-) was also employed in a preliminary study to investigate the stability of NM-300K in medium without serum.

### 2.4. Cell Line Culture

An array of cell lines derived from the rainbow trout were used in this study. RTL-W1, a rainbow trout liver cell line that appears to be derived from biliary preductural epithelial cells [30] was a generous gift from Dr. Bols and Dr. Lee [31]. The RTH-149 rainbow trout hepatoma cell line [32] and RTG-2, a fibroblast-like gonadal cell line [33] were obtained from the American Type Culture Collection (ATTC) (Manassas, VA, USA). The RTL-W1 cell line was cultured in L-15 medium supplemented with 1% L-glutamine and 1% penicillin/streptomycin. Both RTH-149 and RTG-2 cell lines were cultured in EMEM with Earle’s balanced salts and with 1% L-glutamine, 1% penicillin/streptomycin and additionally either 1% sodium pyruvate in the case of RTH-149 or 1% NEAA for the RTG-2 cell line. The former and latter were termed EMEM(Pyr) and EMEM(NEAA), respectively. All media were supplemented with 10% FBS. Complete media formulations can be found in Supplementary Information Table 1.

All cell lines were cultured at 20 ˚C and, in the case of RTH-149 and RTG-2 cell lines, under 5% CO_2_ atmosphere. Cell cultures were maintained in 75 cm^2^ culture flasks (Greiner Bio-one, CellStar, Frichenhausen, Germany) and routinely split one to two times per week using 0.5% trypsin/0.02% EDTA. Experiments were carried out on confluent cell monolayers obtained with a seeding density of 2.5 × 104 cells in 100 µL of culture media after 24 h of culture in 96-well plates (Greiner-Bio one, CellStar, Spain).

### 2.5. Primary Hepatocyte Isolation and Culture

For hepatocyte isolation, juvenile rainbow trout weighing on average 210 ± 5 g and measuring 22 ± 4 cm in length were used. Trout were sacrificed using MS-222 (150 mg/L). A two-step perfusion method and collagenase digestion was used followed by mechanical dissociation of the liver tissue [34,35,36]. Following isolation, hepatocytes were seeded in 24 well culture plates (TPP, Trasadingen, Switzerland) at an optimum seeding density of 50 × 10^4^ cells/ well (400 µL). The primary trout hepatocytes were cultured at 16 ˚C for 24 h in M199 medium supplemented with 10% FBS prior to exposures.

### 2.6. Exposures

Values of LD50 and IC50 reported in the literature after exposure of rainbow trout and rainbow trout cell lines to AgNPs range between 2.5 and 31 µg/mL [16,37]. To cover this reported range of concentrations, the highest and lowest concentrations chosen in the present study were 93.5 µg/mL and 0.73 µg/mL. Taking into account the possibility that cells could show a different sensitivity to the Ag ion than to AgNPs, a wider concentration range was chosen for the Ag ion going from 0.0345 µg/mL Ag to 345 µg/mL Ag. The NM-300K original dispersion was diluted 1/1000 in the corresponding culture medium (depending on cell line/primary hepatocytes) to create the highest exposure concentration (93.5 µg/mL). From this, a 1/2 serial dilution produced the exposure concentration range used: 0.73 to 93.5 µg/mL. In these experiments, exposures were also performed with AgNO_3_ which acted as a reference Ag+ ion source to compare with nanoparticle effects. For AgNO_3_, exposures were performed using a 1/10 serial dilution of the highest exposure concentration, 345 µg/mL of Ag, until reaching the lowest exposure concentration of 0.0345 µg/mL of Ag. For ease of interpretation and comparative purposes, AgNO_3_ exposure concentrations are presented with their respective µg/mL of silver content. Suspensions were also prepared of the dispersant (NM-300KDIS) in culture medium using initially a 1/1000 dilution to mimic the method used to create the highest NM-300K exposure concentration, and then diluted using a factor of 2. Cells exposed to dispersant only acted as a vehicle control in all assays. Cell lines and primary hepatocytes were exposed for 24 h before cytotoxicity assays were performed. This time frame was considered as appropriate to determine cytotoxicity allowing comparisons among cell lines and assay systems.

### 2.7. Cytotoxicity Assessment Using AlamarBlue, CFDA-AM and Neutral Red Uptake (NRU) Assay

We have employed a 3 in 1 fluorometric-based assay system that incorporates a 96-well plate layout and facilitates the simultaneous use of three assays to monitor different endpoints of cytotoxicity following 24 h exposure to AgNO_3_ and NM-300K [38]. AlamarBlue, a CFDA-AM probe and neutral red dye were applied to the same set of cells to monitor metabolic activity, plasma membrane integrity and lysosome functionality, respectively. Cells were first treated with 100 µL of an alamarBlue/CFDA-AM working solution (1.25% v/v AlamarBlue and 4 µM CFDA-AM) prepared in MEM(-) for 30 min under dark conditions. Fluorescence intensity of the AlamarBlue and CFDA-AM conversion products, resorufin and carboxyfluorescin, respectively, were measured at 532 and 590 nm or 485 and 535 nm excitation (exc.) and emission (em.) wavelengths, respectively, using a microplate reader (Tecan Genios, Tecan Group Ltd., Männedorf, CH). Subsequently, the reagents were removed and cells were washed once with PBS before being treated for 1 h with 100 µL of a NR working solution (0.03 µg/mL) prepared in MEM(-) again under dark conditions. After this incubation period, the NR solution was removed and cells washed with PBS. Any retained NR was extracted using 150 µL of an acidified (1% glacial acetic acid) 50% ethanol/49% Milli-Q water solution and fluorescence was measured at 532 nm exc. and 680 nm em. (Tecan Genios microplate reader).

### 2.8. Interference

In order to assess any potential particle auto-fluorescence or quenching at the fluorescence wavelengths used in the cytotoxicity assays readouts, fluorescence readings of nanoparticle suspensions without cells were taken at 532 nm exc./590 nm em. for the AlamarBlue assay, 485nm exc./535 nm em. for the CFDA-AM assay and 532 nm exc./680 nm em. for the NRU assay. Readings were also taken with exposed cells after two washing steps with PBS to assess if the amount of nanoparticles present after washing (either adhered to the cell surface or taken up) is great enough to cause any potential interference. Furthermore, readings were taken of washed cells suspended in maximum concentrations of assay conversion products (resorufin (1 µM), 5-CF (4µM) and NR (0.03 mg/mL)) prepared in MEM(-) to assess if the concentration of nanomaterial (NM) that remains in/on cells after washing could interfere with assay conversion products fluorescence.

### 2.9. Statistics

Data are represented as the mean ± standard error of the mean (SEM) of at least three independent experiments. In each of these experiments, exposure of cells to each of the concentrations of nanoparticle, the salt or the control medium was performed in triplicate. For all statistical analyses, the SigmaPlot® 12.0 software (Systat Software Inc, San Jose, CA, United States) was used. To calculate IC50 (concentration causing a 50% inhibition with respect to the controls) values, results were fitted to a regression model equation for a sigmoid curve: y = max/[1+e-(x-IC50)/b)] + min, where max is the maximal response observed, b is the slope of the curve and min the minimal response. Normality of the data distributions was checked by means of a Shapiro-Wilk test. The homogeneity of variances was checked automatically by the program. Since the data were normal and homoscedastic, a one-way repeated measures analysis of variance (RM-ANOVA) followed by a Dunnett’s post hoc test was performed, allowing the detection of significant difference from control values and the establishment of the lowest observed effect concentration (LOEC) (*p* < 0.05).

## 3. Results

### 3.1. Physico-Chemical Characterisation

The size distribution of silver nanoparticles in the aqueous dispersion and culture media (MEM(-), L-15, EMEM(pyr), EMEM(NEAA) and M199) was characterised using DLS directly after preparation and after 24 h to check stability (Table 1). Hydrodynamic sizes are presented according to intensity, volume and number weighted distributions taking into account the AgNPs refractive index.

DLS analysis shows that aqueous dispersions of NM-300K (93.45 µg/mL) have a stable but polydisperse size distribution (PdI 0.473) with two distinct populations according to intensity distributions with average hydrodynamic diameters of 6 ± 1 nm and 54 ± 6 nm. However, when volume and number distributions are taken into consideration, NM-300K aqueous dispersions show a population averaging 25 ± 3 nm and 4 ± 1 nm, respectively, with a maximum hydrodynamic diameter. These distributions appear as stable after 24 h. In contrast, in serum free MEM(-), the silver nanoparticles aggregate and sediment out of solution over time. DLS analysis of particles that remain in suspension after 24 h reveal that bigger populations, 467 ± 56 nm in diameter, are present. Volume and number measurements show that, together with these particles (570 ± 30 nm), there are also particles present with maximum hydrodynamic diameters of 29 ± 3 and 24 ± 3 nm, respectively.

**Table 1:** Size distribution of the NM-300K dispersion in the various culture medium suspensions used in exposure studies characterised by dynamic light scattering (size distribution by intensity, volume and number).

NM-300K Suspension (93.5 µg/mL)				Hydrodynamic Size by Intensity					Hydrodynamic Size by Volume		Hydrodynamic Size by Number
Medium Type (Cells)	Temp (˚C)	Cl Ion (mM)	Cysteine */ Cystine Content (µM)	Time (h)	PdI	Z-Av (d.nm ± sd)	Peak 1 (d.nm ± sd) (%)	Peak 2 (d.nm ± sd) (%)	Peak 1 (d.nm± sd) (%)	Peak 2 (d.nm± sd) (%)	Peak 1 (d.nm ± sd) (%)
MilliQ Water MEM(-)	20	−	−	T0	0.473	36 ± 7	6 ± 1 (14)	54 ± 6 (86)	4 ± 1 (98)	25 ± 3 (2)	4± 1 (100)
				T24	0.484	36 ± 10	6 ± 1 (9)	56 ± 3 (91)	4 ± 2 (97)	30 ± 4 (3)	3 ±1 (100)
	20	75.7	99	T0	0.453	44 ± 12	6 ± 1‎ (10)	62 ± 8 (90)	5 ± 1 (95)	30 ±6 (5)	4 ± 1(100)
				T24	0.569	188 ± 72	467± 56 (54)	42 ± 3 (46)	29 ± 3 (90)	570 ±30(10)	24 ±3 (100)
L-15 (RTL-W1 *)	20	88.0	990 *	T0	0.418	34 ± 1	7 ± 1 (8)	53 ± 3 (92)	5.8 ± 1 (93)	28 ± 3 (7)	5 ± 1 (100)
				T24	0.234	209 ±4	254 ± 27 (100)		294 ± 36 (100)		136 ± 13(100)
EMEM (pyr) (RTH-149)	20	75.7	99	T0	0.529	58 ± 2	9 ± 2 (9)	70 ± 9 (91)	7 ± 1 (95)	24 ± 7 (5)	5 ± 2(100)
				T24	0.441	53 ± 1	9 ± 1 (11)	83 ± 2 (89)	8 ± 1 (96)	23 ± 3 (4)	6 ± 1(100)
EMEM (NEAA) (RTG-2)	20	75.7	99	T0	0.455	53 ± 3	8 ± 1 (7)	79 ± 7 (93)	5 ± 1 (97)	21 ± 7 (3)	4 ± 1 (100)
				T24	0.297	43 ± 1	7 ± 2 (5)	64 ± 7 (95)	6 ± 2 (92)	26 ± 8 (8)	5 ± 2 (100)
M199 (primary hepatocytes)	16	75.3	5 * /83	T0	0.430	35 ± 1	6 ± 2 (6)	58 ± 3 (94)	5 ± 1 (92)	26 ± 6 (8)	4 ± 1 (100)
				T24	0.428	35 ± 1	6 ± 1 (8)	58 ± 4 (92)	5 ±1 (96)	26 ± 2 (4)	4 ± 1 (100)
Mean size distribution				T0	0.458	45 ± 12	7.5 ± 1 (7)	65 ± 12 (93)	6 ± 1 (94)	24 ± 3 (6)	5 ± 1 (100)
* excluding RTL-W1 culture medium L-15				T24 *	0.389	44 ± 9	7.3 ± 2 (8)	68 ± 13 (92)	6 ± 2 (95)	25 ± 2 (5)	5 ± 1 (100)

In L-15 medium, visual observation confirmed evidence of sedimentation and darkening of the NM-300K suspension (Supplementary Materials Figure S1). DLS measurements considering intensity readouts in this medium showed particles in suspension 254 ± 27 nm with average diameter. There was no evidence of smaller populations even when representing distributions in terms of volume or number. A PdI value of 0.234 confirmed that the silver nanoparticles are present in single populations of bigger sizes in this medium. With the exception of L-15, in all complex culture media (*i.e.*, EMEM (pyr), EMEM(NEAA) and M199) used for the cell lines and primary hepatocytes exposures the NM-300K dispersion is stable over 24 h and the frequency size distribution showed no important differences among media (see Table 1 for all details). Taking the mean size distribution for all medium suspensions, hydrodynamic diameters and respective intensity percentages for the populations are 7.5 ± 1 nm (7%) and 65 ± 12 nm (93%) in intensity distribution, 6 ± 1 nm (94%) and 24 ± 3 nm (6%) for volume distribution and 5 ± 1 nm (100 %) in number distribution. Very similar values are obtained after 24 h of incubation (Table 1). Size distribution frequency curves of the NM-300K dispersion in the different complex culture medium suspensions after 24 h incubation can be found in Supplementary Materials Figure S2.

### 3.2. Interference

The NM-300K dispersion in cell free suspensions had no fluorescence emission on their own. However, concentration dependent reductions in fluorescence units were seen due to both solution turbidity and possible physical interference from particles sedimented out of solution in the case of L-15 culture medium (see Supplementary Materials Figure S3). When NM-300K suspensions were incubated with cells under exposure assay conditions, and the washing steps were applied as in the cytotoxicity assays, the quantity of NM that remained in both stable (EMEM (pyr), EMEM (NEAA), M199) and sedimented (L-15) suspensions was not great enough to interfere with the fluorescent signal or mask the fluorescent product (resorufin, 5-CF or NR dye) signal.

### 3.3. Cytotoxicity of AgNO_3_ and NM-300K

Dispersant alone (NM-300KDIS) at the concentrations corresponding to the exposure assays did not provoke any cytotoxic effect in any of the assay systems used.

#### 3.3.1. AlamarBlue Assay

**Figure 1:**
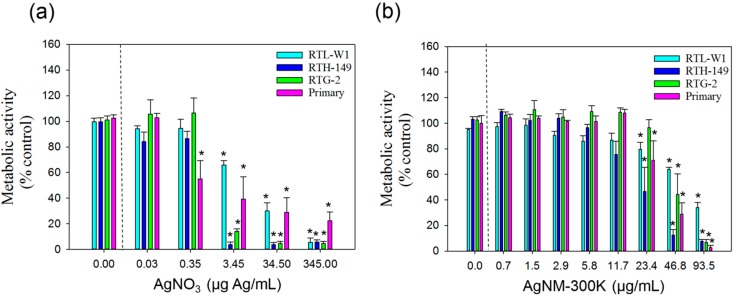
Cytotoxicity of AgNO_3_ and AgNPs NM-300K according to AlamarBlue assay. (**a**) Cytotoxicity measured using the AlamarBlue assay and metabolic activity as endpoint of toxicity in liver cell lines (RTL-W1 and RTH-149), a gonadal cell line (RTG-2) and primary hepatocytes from rainbow trout following 24 h exposure to AgNO_3_; (**b**) silver nanomaterial NM-300K. Cells exposed to concentration 0 received the maximal concentration of the dispersant and served as control. Values represent the mean ± standard error of the mean (SEM) (n = 3); * denotes statistically significant differences from control (*p* < 0.05).

The cytotoxicity results obtained with this assay for all cell lines and primary hepatocytes following exposure to AgNO_3_ and NM-300K are presented in Figure 1 and Table 2. The IC50 values following 24 h exposure to AgNO_3_ and NM-300K are presented in (Table 2). For AgNO_3_, the RTL-W1 cell line exhibited the highest IC50 value (10.9 µg/mL) while the other cells used in these experiments exhibited much lower IC50 values (1.1–2 µg/mL). Taking LOEC values into account, the primary hepatocytes showed increased sensitivity (LOEC = 0.34 µg/mL) with respect to the other cell lines (Figure 1 (a)).

**Table 2:** IC50 (µg /mL) values calculated from the different cytotoxicity assay systems in the rainbow trout cell lines and primary hepatocytes after exposure to AgNO_3_ and NM-300K.

Assay system	AlamarBlue			CFDA-AM		NRU	
	AgNO_3_	NM-300K		AgNO_3_	NM-300K	AgNO_3_	NM-300K
Cell line	IC_50_ (µg/mL)						
*RTL-W1*	11	75.9		32.2	15.9	10.9	10.7
*RTH-149*	1.1	19.8		1.4	21.8	0.4	24.9
*RTG-2*	2.8	41.7		1.3	43.1	1.0	37.2
*Primary hepatocytes*	2.0	30.6		2.7	37.7	3.8	45.2

After exposure to NM-300K, the RTL-W1 cell line showed higher IC50 values (75.9 µg/mL) than the other cells (19.8 µg/mL to 41.7 µg/mL). Primary hepatocytes, RTH-149 and RTL-W1 exhibited similar LOEC values (23.4 µg/mL). The LOEC value recorded for the RTG-2 gonadal cell lines was 46.8 µg/mL (Figure 1 (b)).

#### 3.3.2. CFDA-AM Assay

**Figure 2:**
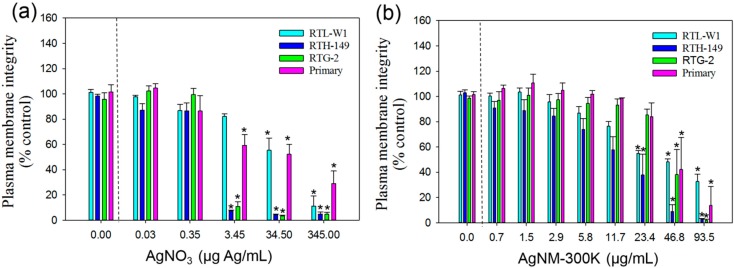
Cytotoxicity of AgNO_3_ and AgNPs NM-300K according to CFDA-AM assay; (**a**) Cytotoxicity measured using the CFDA-AM assay and plasma membrane integrity as endpoint of toxicity in liver cell lines (RTL-W1 and RTH-149), a gonadal cell line (RTG-2) and primary hepatocytes from rainbow trout following 24 h exposure to AgNO_3_; (**b**) silver nanomaterial NM-300K. Cells exposed to concentration 0 received the maximal concentration of the dispersant and served as control. Values represent the mean ± standard error of the mean (SEM) (n = 3); * denotes statistically significant differences from control (*p* < 0.05).

Curves showing concentration dependent effects obtained with this assay are presented in Figure 2, whereas IC50 values of these curves are shown in Table 2. Again, the RTL-W1 cell line had the highest IC50 and the LOEC value for the RTL-W1 cell line following AgNO3 exposure was 10-fold higher (34.52 µg/mL) than for primary hepatocytes, RTH-149 and RTG-2 cell lines (3.45 µg/mL) (Figure 2 (a)).

In contrast to responses to AgNO_3_, the RTL-W1 cell line showed the lowest IC50 value following NM-300K exposure (Table 2). Thus, according to IC50 values in this assay, the cell sensitivity for NM-300K toxicity follows the order: RTL-W1 > RTH-149 > Primary hepatocytes > RTG-2. Both liver cell lines (RTH-149 and RTL-W1) showed lower LOEC values (23.4 µg/mL) than primary hepatocytes and RTG-2 cells (46.8 µg/mL) (Figure 2(b)).

#### 3.3.3. Neutral Red Uptake Assay

**Figure 3:**
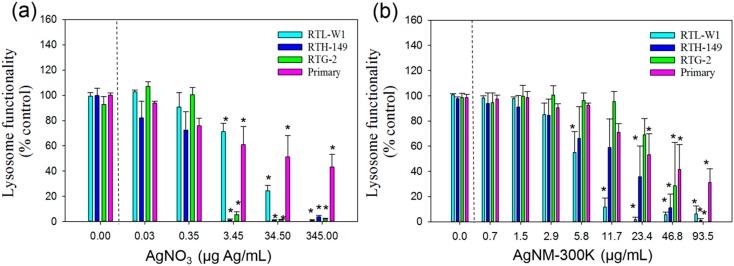
Cytotoxicity of AgNO_3_ and AgNPs NM-300K according to NRU assay; (**a**) Cytotoxicity measured using the NRU assay and lysosome functionality as toxicity endpoint in liver cell lines (RTL-W1 and RTH-149), a gonadal cell line (RTG-2) and primary hepatocytes from rainbow trout following 24 h exposure to AgNO3; (**b**) silver nanomaterial NM-300K. Cells exposed to concentration 0 received the maximal concentration of the dispersant and served as control. Values represent the mean ± standard error of the mean (SEM) (n = 3); * denotes statistically significant differences from control (*p* < 0.05).

Concentration response curves according to this assay system for the cell lines and hepatocytes following exposure to AgNO_3_ and NM-300K are presented in Figure 3. Similarly to the previous assays, the highest IC50 value for AgNO_3_ was recorded for the RTL-W1 cell line but all the fish cells showed a NOEC of 3.45 µg/mL (Figure 3(a)).

For the AgNP, the RTL-W1 was the most sensitive showing lower IC50 and LOEC values than the other cells (Table 2 and Figure 3(b)). The LOEC value for the RTL-W1 cell line was 5.8 µg/mL according to the NRU assay (Figure 3(b)). In contrast, the RTG-2 cell line showed the lowest sensitivity towards NM-300K exposure according to a recorded LOEC value of 46.8 µg/mL.

## 4. Discussion

In this study, we have tested the toxicity of AgNO_3_ and AgNPs (using the representative silver nanomaterial NM-300K from the JRC Nanomaterials Repository) towards rainbow trout liver cell lines (RTL-W1, RTH-149), a gonadal cell line (RTG-2) and primary hepatocytes. For chemicals in general, *in vitro* systems are still being validated and their regulatory applicability is still being explored, taking into account, in particular, their value in predicting *in vivo* responses. In the case of NMs, that possibly present additional confounding factors, the use of *in vitro* systems raises new concerns. We sought to address these issues.

Prior to nanoparticle cytotoxicity assessment, it is essential to take into consideration the possible interference between *in vitro* assay systems and nanomaterials [39,40,41,42]. In this work, this issue was carefully assessed and it was shown that the amount of NM-300K that remained inside or adhered to the cells was not great enough to cause interference with the used cytotoxicity assays.

According to DLS analysis, NM-300K suspensions prepared in EMEM (RTH-149, RTG-2 cells) and M199 (primary hepatocytes) were stable over the 24 h exposure period. Taking volume distributions as the most accurate representation, due to intensity masking of smaller particles and number distributions favouring smaller particles, the NM-300K size distribution in culture medium is composed of 95% of particles with an average diameter of 7 ± 1 nm and 5% 25 ± 2 nm in diameter. This guarantees a real exposure to nanoparticulate material. All media were supplemented with FBS which appeared to ensure stability. In contrast to other media suspensions, particles prepared in L-15 medium (RTL-W1 cells) experienced aggregation formation and precipitated out of solution over the 24 h exposure timeframe. L-15 has high levels of thiol containing amino acids including cysteine and methionine which are strong Ag+ complexing ligands known to form Ag–S covalent bonds with AgNPs. It also has a higher chloride content that could favour the formation of silver chloride compounds that could precipitate out of solution [43]. Therefore, although L-15 is widely used as a cell culture medium and has been used previously in nanoparticle toxicity studies with fish cells [19,44] results of this study would suggest that it should be applied with maximal caution in these kinds of approaches. As seen in this study and also highlighted by Yue and colleagues [45], the choice of cell culture medium can affect AgNP physico-chemical behaviour and thus is likely to also have a direct effect on toxicity.

In this study, AgNO_3_ was included as a Ag+ ion source to investigate differences in the toxicity of Ag+ ions and AgNPs and also if differences in sensitivities to Ag+ exist between different cell lines and hepatocytes. Previous studies with CuNPs and ZnO NPs demonstrated an increased sensitivity of mammalian cell lines with respect to fish cell lines to nanoparticle toxicity, possibly due to differences in their sensitivity to ions released from the nanoparticles rather than to nanoparticle effects [46,20]. There is also debate surrounding the influence of Ag+ ion on nanoparticle toxicity and conflicting results showing either that nanoparticles are more or less toxic than equivalent concentrations of Ag+ [47,48]. In this study, in general, cytotoxic effects were observed at lower concentrations of AgNO_3_ than NM-300K, which suggests a higher toxicity of ions.

IC50 values corresponding to AgNO_3_ for RTH-149, RTG-2 and primary hepatocytes ranged from 0.4 to 3.8 µg/mL across all toxicity endpoints. This is in agreement with Farkas and colleagues [49] who showed an IC50 of 1.1 µg/mL following AgNO_3_ exposures using rainbow trout primary hepatocytes and the AlamarBlue assay system. In contrast, the RTL-W1 liver cell line was distinctly the least sensitive, and the toxic potency of AgNO_3_ was around 10-fold lower (IC50 ranging from 10.9 to 32.2 µg/mL) than in the rest of the cells used in this study. As previously discussed, the difference in sensitivity and susceptibility in this cell line is most likely related with decreased bioavailability and complexing of the Ag+ ions in L-15 medium. This important observation has also been made by Dayeh and colleagues [50] when using L-15 medium and testing the toxicity of copper sulphate (CuSO_4_) and copper chloride (CuCl_2_) in fish cells, where toxicity was absent but appeared when using amino acid free minimal medium (L15/ex). This draws attention to the important influence of medium composition on metal ion toxicity in cell lines *in vitro*. Kermanizadeh and co-authors reported IC50 values of 14.06 and 31.25 µg/mL for NM-300K toxicity in human renal proximal tubule epithelial cells (HK-2) following exposure in different mediums [51]. A similar phenomenon has been reported for AgNP toxicity studies *in vivo* towards rainbow trout fry with LOEC values ranging from 0.7 to 25 µg/mL, increasing with increasing water salinity [52]. Therefore, medium composition/exposure environment can be a key factor not only in influencing particle behaviour but in dictating toxicity.

A concentration dependent reduction in viability following exposure to NM-300K (0.73 to 93.5 µg/mL) in all cell lines and primary hepatocytes was observed. IC50 values ranged from 10.7 to 75.9 µg/mL according to the specific cell line and toxicity assay endpoint used. Despite its apparent increased resistance to AgNO_3_, the RTL-W1 cell line proved particularly susceptible to toxic effects of NM-300K when measured as lysosomal damage (NRU assay, with the lowest IC50 value recorded: 10.7 µg/mL) or membrane disruption (CFDA-AM assay, with an IC50 of 15.9 µg/mL). However, the IC50 values according to the AlamarBlue assay were the highest recorded (IC50 75.9 µg/mL). Similarly Gaiser and co-authors [53] showed lower IC50 values using the lactate dehydrogenase (LDH) leakage assay which measures loss of membrane integrity compared to the AlamarBlue assay (8 µg/mL *vs.* 64 µg/mL respectively) using the same NM-300K particles in the human hepatic cell line C3A. This may point to a specific mechanism of toxicity for the NM-300K suspensions targeting membranes, particularly those of lysosomes. It is possible that the bigger aggregates present in RTL-W1 culture medium (L-15) can cause lysosomal damage at lower exposure concentrations. In fact, lysosomal perturbation has been reported for a variety of NMs (reviewed in [54]) and is seen as an emerging mechanism of NM toxicity. According to a study by Yue and colleagues [45], using the RTgill-W1 rainbow trout gill cell line, lysosomal membrane integrity was also a significantly more sensitive toxicity endpoint for citrate capped AgNPs. Furthermore, they have also shown that cysteine can protect against metabolic activity and membrane damage but not lysosomal damage in the case of AgNPs, thus corroborating our findings in this study. This could be due to different uptake mechanisms of these aggregated AgNPs and, while outside of the scope of this study, this warrants further investigation.

When comparing the responses of the continuous cell lines to the primary hepatocytes in general primary cells showed comparable sensitivity both to Ag+ and AgNPs. However, when LOEC values were taken into account, primary cells showed increased sensitivity to Ag+ according to the AlamarBlue assay system which measures metabolic activity. The lack of any noticeable difference in the physico-chemical properties of AgNPs in media used for primary hepatocyte exposures suggests that the exposure environment did not contribute to this increased sensitivity. Primary cells exhibit metabolic pathways and capacities quite similar to the cells in original tissues, while continuous cell lines have lost some of them. This can be associated with higher detoxification capabilities (and therefore lower sensitivity to toxic action of substances) but also with higher sensitivities (to the toxic action of substances) due to alterations of any of the very closely related metabolic pathways or due to a favored uptake rate of Ag+. Any or a combination of these factors could explain the differences in sensitivity that would need to be addressed in future studies. Interestingly, the increased sensitivity is only seen following exposure to AgNO_3_ and not when exposed to AgNPs suggesting that possessing full metabolic processes (e.g. cytochrome P450–mediated oxidation) may not be particularly significant for the toxicology of NPs. This point has also been made by other authors [53] when comparing responses to AgNPs in rat liver models *in vitro* and *in vivo*.

*In vitro* studies are always limited with respect to *in vivo* approaches by not being able to represent different tissue specific toxic insults, adaptive responses at whole organism levels and toxicokinetics [55]. As a consequence, they show in general lower sensitivity than *in vivo* systems [56,57]. The recently reported LC50 value in juvenile rainbow trout following 48 h AgNP exposure was 3.13 µg/mL [16]. In our study, IC50 values ranged from 10.7 to 75.9 µg/mL, representing three to 24-fold higher values than that reported in *in vivo* LC50. Apart from the obvious metabolic and complexity differences between *in vitro* and *in vivo* systems, one factor that helps to explain the higher IC50 values is that the actual concentrations of bioavailable AgNPs in this study are most likely much lower due to the presence of serum proteins in the culture media. Massarsky and co-authors, also using rainbow trout primary hepatocytes, only report subtle responses to 31 µg/mL of AgNPs when bovine serum albumin was present in the exposure medium [37]. In contrast, studies using serum free medium recorded IC50 values between 2.5 and 4.9 µg/mL in rainbow trout primary hepatocytes [49]. In our study, we have used serum due to its physiological relevance and to reflect conditions in the circulatory environment which the nanoparticles would be exposed to prior to distribution in the liver. Incorporation of FBS also ensured the exposure of cells to stable AgNP preparations. All this must be taken into account in the future development of standard guidelines for *in vitro* tests in order to increase the predictive value of *in vitro* studies for *in vivo* responses. For now, working with well-defined representative NMs, choosing cell lines from relevant target tissues and using a battery of cytotoxicity assays, as in the present study, can be very useful in toxicity ranking and as pre-screening to help regulators in making the decision to avoid particular *in vivo* assays. It is also evident that the applied approach represents an invaluable tool for mechanistic studies.

## 5. Conclusions

Up until now, the cytotoxicity of the NM-300K silver representative nanomaterial has not been tested in liver cells of fish. In this comparative study, liver cell lines (RTH-149, RTL-W1) proved as sensitive test systems when compared to primary hepatocytes following exposure to NM-300K. Considering the ease of maintenance and culture of cell lines compared with primary hepatocytes, this has important practical implications for the integration of such a tool into intelligent testing strategies for the hazard assessment of nanoparticles. However, further work is necessary to determine the applicability of this tool to other NPs using other cellular systems that could be more representative of particular conditions or environments.

Using the simultaneous application of three different assay systems (AlamarBlue, CFDA-AM and NRU) proved a valuable means to assess cytotoxicity according to different targets of toxic action. The lowest IC50 was recorded using the NRU assay and the RTL-W1 cell line (10.7 µg/mL) and, thus, points to lysosomal damage as an important indicator for detecting nanoparticle specific effects. It must be noted, however, that cell culture medium composition had a strong influence on the behaviour and toxicity of Ag+ and AgNPs, causing precipitation or limiting their bioavailability and leading to a reduction of or increase in toxicity according to particular tests applied. Therefore, while such an *in vitro* testing approach was easily applicable careful consideration must be made regarding the influence of cell culture medium composition when using cellular *in vitro* systems for observing toxicity of NPs. Choosing culture media with similar compositions for exposures will ensure the same NM presentation to respective cell lines being tested and facilitate an accurate comparative toxicity assessment.

## Supplementary Files

Supplementary File 1
