# Supplementary material for: Comparative Cytotoxicity Study of Silver Nanoparticles (AgNPs) in a Variety of Rainbow Trout Cell Lines (RTL-W1, RTH-149, RTG-2) and Primary Hepatocytes
Source: Int J Environ Res Public Health. 2015 May 20;12(5):5386–405. doi: 10.3390/ijerph120505386 (PMC4454974; doi:10.3390/ijerph120505386)
Supplement: Supplementary File 1 [file ijerph-12-05386-s001.pdf]

Comparative Cytotoxicity Study of Silver Nanoparticles (AgNPs) in a Variety of Rainbow Trout Cell Lines (RTL-W1, RTH-149, RTG-2) and Primary Hepatocytes

---

Table S1. Composition of L-15, EMEM(pyr), EMEM(NEAA) and M199 complex culture media used to prepare AgNM-300K suspensions and expose the cell lines and primary hepatocytes.

**Cell Line**	**RTL-W1**	**RTH-149**	**RTG-2**	**Primary Hepatocytes**
Cell culture medium	Leibovitz L-15	EMEM (pyr)	EMEM (NEAA)	M199
Component	Concentration (mg/L)			
Inorganic Salts				
Calcium chloride (CaCl2) (anhyd.)/(dihyd)	140 (anhyd.)	265 (dihyd.)	265 (dihyd.)	200 (anhyd.)
Potassium chloride (KCL)	400	400	400	400
Sodium chloride (NaCL)	8000	6800	6800	6800
Ferric nitrate (monohyd.)	–	–	–	0.720
Magnesium chloride	93.67	–	–	–
Magnesium sulphate (anhyd.)	97.70	200	200	97.67
Sodium acetate (anhydr.)	–	200	200	50
Potassium phosphate (KH_2PO_4)	60			–
Sodium phosphate (NaH_2PO_4) (monohyd.)/(anhyd.) *	190 *	140	140	140
Sodium bicarbonate ($NaHCO_3$)	–	2200	2200	–
Sodium pyruvate	550	110 (Added)	–	–
Amino Acids				
D/L-Alanine	450	–	8.9 (Added)	25
L-Arginine/L-Arginine HCL *	500	126.4 *	126.4 *	70
L-Asparagine	250	–	13.21 (Added)	–
L-Aspartic acid	–		13.30 (Added)	30
L-Cysteine/L-Cysteine HCL (monohyd.) *	120	24	24	26 *
L-Glutamine	292 (Added)	292	292	100
L-Glutamic acid	–	–	14.7 (Added)	67
Glycine	200	–	7.5 (Added)	50
L-Histidine/L-Histidine HCL (monohyd.) *	250	42 *	42 *	21.88 *

Table S1. *Cont.*

**Cell Line**	**RTL-W1**	**RTH-149**	**RTG-2**	**Primary Hepatocytes**
D/L-Isoleucine	250	52.4	52.4	20
L-Leucine	125	52.4	52.4	60
L-Lysine/L-Lysine HCL *	75	73 *	73 *	15 *
D/L-Methionine	150	15	15	25
D/L-Phenylalanine	250	33	33	25
L-Serine	200	–	19.5 (Added)	25
L-Proline/Hydroxy L-Proline *	–	–	11.5 (Added)	40/10 *
D/L-Threonine	600	47.6	47.6	30
L-Tyrosine/L-Tyrosine 2Na (dihyd.) *	370 *	36.2	36.2	57.66 *
L-Tryptophan	20	10.2	10.2	10
D/L-Valine	200	46.8	46.8	25
Vitamins				
Ascorbic acid				0.05
Biotin				0.1
Choline chloride	1	1	1	0.5
D/L-Ca Pantothenate	1	1	1	0.01
Ergocalciferol	–			0.1
i-inositol	2	2	2	0.05
Folic acid	1	1	1	0.01
Menadione	–			0.01
Niacin	–			0.025
Nicotinamide	1	1	1	0.025
4-Aminobenzoic acid (PABA)	–			0.05
Pyrridoxal HCL	–			0.025
Pyridoxine HCL	1	1	1	0.025
Riboflavin/Riboflavin -5-PO_4 Na *	0.1 *	0.1	0.1	0.01
Thiamine HCL/Thiamine monophosphate *	1 *	1	1	0.01
DL-α-tocopherol phosphate 2Na	–	–	–	0.01
Vitamin A acetate	–	–	–	0.14
Other components	–	–		
Adenine Sulphate (dihydr.)	–	–	–	10.98
Adenosine 5’-monophosphate (AMP) (monohyd.)	–	–	–	0.2
Adenosine 5’-triphosphate (ATP) 2Na (trihyd.)	–	–	–	1.098
Cholesterol (synthetic)	–	–	–	0.2
2-deoxy-d-ribose	–	–	–	0.5
Dextrose	–	–	–	1000
L-Glutathione (reduced)	–	–	–	0.05

Table S1. *Cont.*

**Cell Line**	**RTL-W1**	**RTH-149**	**RTG-2**	**Primary Hepatocytes**
Guanine HCL (monohyd.)	–	–	–	0.3
Hypoxanthine Na	–	–	–	0.345
D-Ribose	–	–	–	0.5
Thymine	–	–	v	0.3
Tween 80	–	–	–	20
Uracil	–	v	–	0.3
D(+)Galactose/Glucose	900	1000	1000	1000
Phenol Red Na	10	10	10	21.24
Penicillin	10000 U/mL	10000 U/mL	10000 U/mL	–
Streptomycin	10	10	10	–
Xanthine Na	–	–	–	0.344
Serum				
Fetal bovine serum (FBS)	10% (Added)	10% (Added)	10% (Added)	10% (Added)

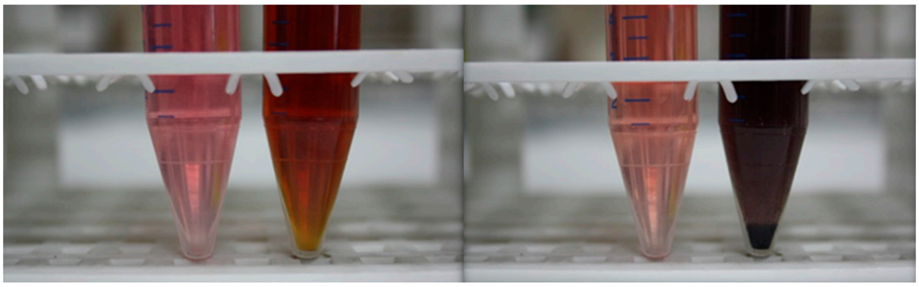

(**a**) (**b**)

Figure 1. Contrasting appearance of NM-300K suspensions (93.45 µg/mL)(right side) prepared in EMEM culture medium (**a**) and L-15 culture medium (**b**) compared to respective medium only (left side).

(a)

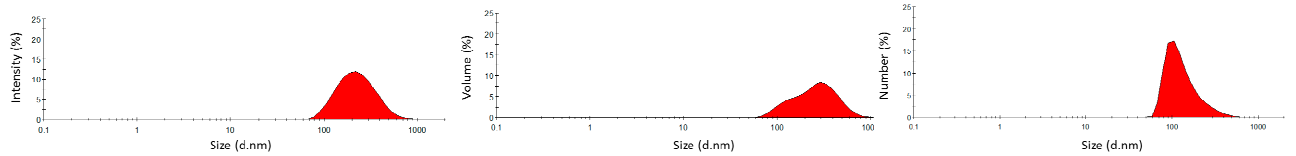

(b)

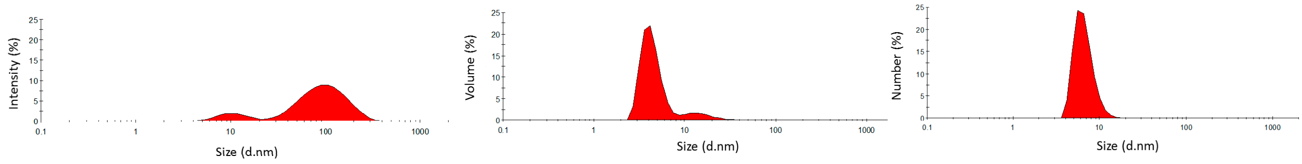

Figure 2. *Cont.*

(c)

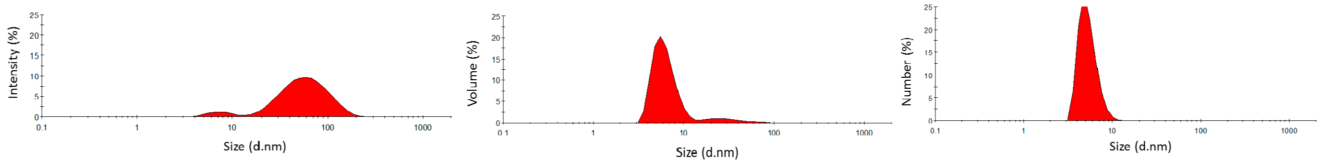

(d)

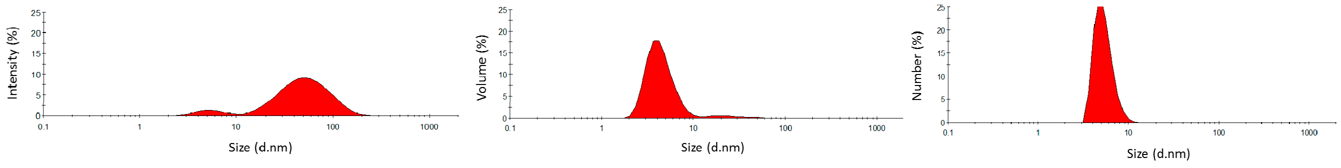

Figure 2. DLS size distribution frequency curves of NM-300K dispersion (93.45 µg/mL) in different complex culture media suspensions after 24 h incubation; L-15 culture medium (RTL-W1) (**a**), EMEM(pyr) culture medium (RTH-149) (**b**), EMEM(NEAA) culture medium (RTG-2) (**c**), and M199 culture medium (Primary hepatocytes) (**d**). Size is measured using diameter in nanometers (d.nm) and profiles are presented as intensity, volume and number distributions (left, centre and right).

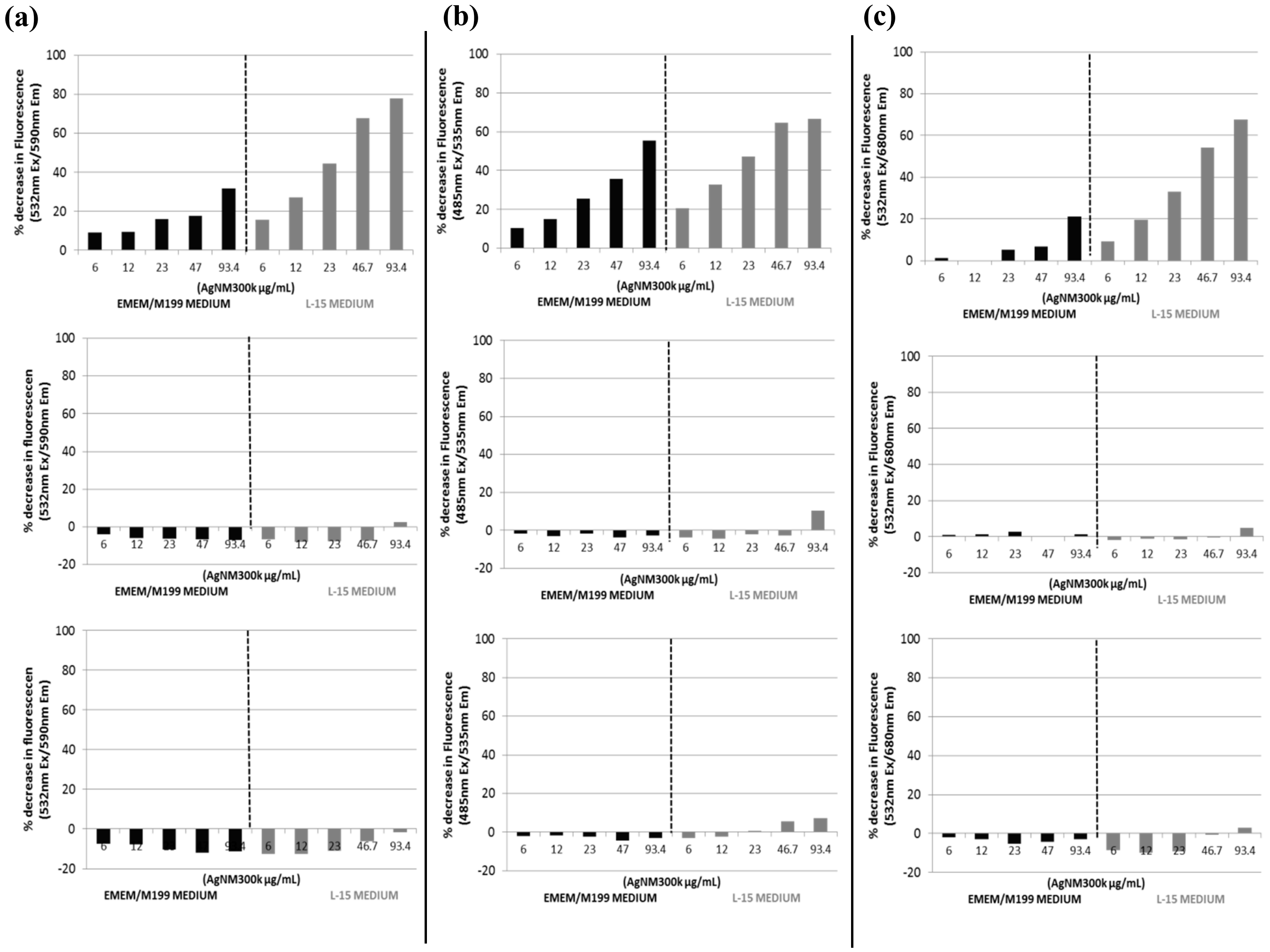

Figure 3. Investigating the potential interference of exposure concentration range of NM-300K suspensions (5.8 to 93.45 µg/mL) prepared in EMEM/M199 culture medium and L-15 culture medium with assay fluorescence readouts of AlamarBlue (**a**), CFDA-AM (**b**) and NRU (**c**) assays. Top: Cell-free suspensions following 24 h incubation in either EMEM/M199 or L-15 medium. Middle: Suspensions with cells following 24 h incubation in either EMEM/M199 or L-15 medium followed by washing steps. Bottom: Cells after wash step with PBS in presence of assay products (resorufin, CF, NR dye according to alamarBlue, CFDA-AM and NRU assay respectively). Interference is represented as % decrease in fluorescence compared to medium suspension only or assay products at respective wavelengths.

© 2015 by the authors; licensee MDPI, Basel, Switzerland. This article is an open access article distributed under the terms and conditions of the Creative Commons Attribution license (http://creativecommons.org/licenses/by/4.0/).
